# GaitDynamics: A Generative Foundation Model for Analyzing Human Walking and Running

**DOI:** 10.21203/rs.3.rs-6206222/v1

**Published:** 2025-03-21

**Authors:** Tian Tan, Tom Van Wouwe, Keenon F. Werling, C. Karen Liu, Scott L. Delp, Jennifer L. Hicks, Akshay S. Chaudhari

**Affiliations:** 1 Department of Radiology, Stanford University; 2 Department of Bioengineering, Stanford University; 3 Department of Computer Science, Stanford University; 4 Department of Mechanical Engineering, Stanford University; 5 Department of Biomedical Data Science, Stanford University

## Abstract

Understanding the dynamics of human gait, including both motions and forces, is vital to promote human health and performance. Conventional gait analysis requires laboratory-based experiments and physics-based simulations to quantify gait dynamics and analyze how dynamics change with treatment, training, injury, and disease. However, the high costs associated with experiments and simulations has confined the use of gait dynamics to small-scale research studies. While deep learning models offer low-cost prediction, and can be highly expressive in fitting large-scale data, existing models have primarily been trained on small datasets with homogenous demographics and focused on predicting a single output. To overcome these limitations, we developed GaitDynamics, a generative foundation model for human gait that is trained on a large dataset with diverse participant demographics and gait patterns. GaitDynamics can be used for diverse tasks with different inputs, outputs, and clinical applications, which we illustrate in three examples: i) estimating ground reaction forces from kinematics with high accuracy and robustness even with missing kinematic data and for populations not included in the training dataset, ii) predicting the influence of gait modifications on knee loading without the need for resource-intensive experiments, and iii) predicting kinematic and force changes that occur with increasing running speeds. These representative tasks demonstrate that GaitDynamics makes accurate and rapid predictions in seconds based on flexible inputs, showing its potential to assess and optimize gait for injury prevention, disease treatment, and performance coaching. All data, code, and trained models are publicly shared.

## Introduction

Quantifying the dynamics of walking and running, including the motions of the body segments and the joint moments that drive the motion, can help diagnose disease, prevent injury, customize rehabilitation, and improve athletic performance. For example, the magnitude, symmetry, and variability of gait parameters such as ground reaction forces can provide insights into the mechanisms of sports injury^1,2^ as well as the rehabilitation status of patients with conditions such as stroke^3,4^, patellofemoral pain^5^, and ligament tears^6^. Measurement of joint loading during walking has also helped develop and personalize gait training interventions to mitigate knee osteoarthritis^7–10^. Understanding the relationship between running speeds, kinematics, and kinetics can enable more effective coaching to optimize human performance^11,12^.

Traditionally, gait dynamics are measured in specialized laboratories, where trained personnel operate expensive systems to measure motion and ground reaction forces. The data collection process often requires several hours of expert time for even a single participant, which can be particularly burdensome for participants with mobility limitations. Physics-based simulations, where human dynamics are optimized to match experimental data or hypotheses about the motion, have been applied in many studies to analyze and predict gait dynamics^13,14^. Prior studies have used simulations to estimate kinetic parameters based on measured kinematics, eliminating the need for force plates that are unavailable outside of specialized laboratories^15–17^. Simulations have also helped identify the influence of gait modifications on joint moments^18,19^. Although simulations reduce or eliminate the need for laboratory-based experiments, they also can be time-consuming and rely on specialized expertise, often requiring hundreds of hours for a single seconds-long simulation. To mitigate the long computation time, many studies have resorted to simplified human models, for example, constraining the simulation in the sagittal plane to reduce degrees of freedom^15,20^. The time-consuming nature of human experiments and simulations limit both the widespread assessment of gait parameters and the discovery of effective gait modification strategies across diverse populations who may benefit from such studies.

Many data-driven models have been proposed for estimating gait parameters such as ground reaction forces^21–25^. While such models can achieve real-time speed and high accuracy, several limitations hinder their applicability in real-world environments. First, these models are designed solely to estimate the unmeasured gait parameters, but none predict how dynamics change due to modifications of specific gait parameters. The ability to predict the dynamic outcomes of modifications (e.g., increasing speeds or medial-lateral trunk sway) could potentially enable the discovery of strategies that reduce risky joint loading or enhance performance. Second, the architectures of these models require the inputs to be complete. In practice, the input data can be incomplete due to factors such as infeasibility of sensor placement, data packet drop in wireless transmission, and camera occlusion. A flexible model that can process arbitrary combinations of input data would be robust to real-world conditions where inputs might be incomplete. Third, models are typically trained and evaluated on data collected from a single laboratory, which can limit generalizability to other datasets with diverse cohorts or environments.

In recent years, we have witnessed remarkable advancements in large computer vision and natural language models^26^. These models are highly flexible in both the inputs they accept and the tasks to which they can be applied, earning them the name, “foundation models”. However, since existing human gait dynamics models are primarily trained on small, proprietary datasets and focus on a specific task, foundation models have not yet emerged for human gait dynamics. One major barrier to the development of foundation models is the availability of large-scale datasets with synchronized kinematics and kinetics for gait. The new AddBiomechanics dataset helps overcome this barrier^27^. The dataset harmonizes movement data from 15 studies, aggregating more than 70 hours of diverse movement data from 270 participants. The size and diversity of AddBiomechanics dataset can enable training a foundation model for gait dynamics that is capable of performing multiple common prediction and analysis tasks.

In this paper, we propose GaitDynamics—a generative foundation model capable of accomplishing multiple common prediction and analysis tasks pivotal to the understanding of gait dynamics. GaitDynamics is trained on the diverse AddBiomechanics dataset, which contains a range of speeds and gait patterns. Our model represents several notable advancements over previous deep learning models in gait analysis. First, the model supports analysis tasks beyond estimating unmeasured gait parameters, such as quantifying how dynamics change with increasing running speeds or modified gait patterns. Second, the model can estimate ground reaction forces based on a flexible combination of input kinematics. Third, the model generalizes to studies not used for training. Notably, despite being trained exclusively on data from healthy participants, GaitDynamics maintains its accuracy amongst individuals with osteoarthritis. GaitDynamics generates a seconds-long trial within one second, which is a thousand-fold more efficient than physics-based predictive simulation that can take anywhere from 35 minutes to hundreds of hours^18–20,28–30^. To facilitate broad usage and customizations of GaitDynamics, we have open sourced the trained model and code.

## Results

### An overview of GaitDynamics

GaitDynamics models full-body kinematics, including pelvis linear velocities with respect to the Earth (hereafter referred to as velocities for simplicity), joint angles, and joint angle velocities, as well as ground reaction forces (hereafter referred to as forces for simplicity) for both feet. The forces include vertical, anterior-posterior, and medial-lateral components, along with the center of pressure—the point where the force acts on the foot. Unlike prior end-to-end models that map a fixed combination of inputs to outputs, GaitDynamics uses flexible combinations of inputs to generate outputs through a diffusion model. Model architecture is detailed in the Methods section. As a foundation model trained on a large, diverse training dataset, GaitDynamics can perform multiple downstream tasks. Here we present three of them: (1) estimating forces using flexible combinations of kinematic inputs, (2) predicting the effects of a trunk sway gait modification on risky knee loading without the need for new human experiments, and (3) predicting a complex set of spatio-temporal, kinematic, and dynamic changes that occur as running speed increases.

### Downstream Task 1: estimating forces using flexible combinations of kinematic inputs

The ability to accurately infer forces during gait from kinematics would transform biomechanics and healthcare, facilitating applications such as remote rehabilitation, exoskeleton control, and running injury prevention. In contrast to prior models that can only use a fixed combination of inputs, GaitDynamics can use flexible combinations of kinematics as inputs. When using partial-body kinematics as input (e.g., without trunk kinematics), GaitDynamics maintained its force estimation accuracy by first using a diffusion model to generate unknown kinematics and subsequently using a refinement model to map full-body kinematics to forces.

**Fig. 1.**
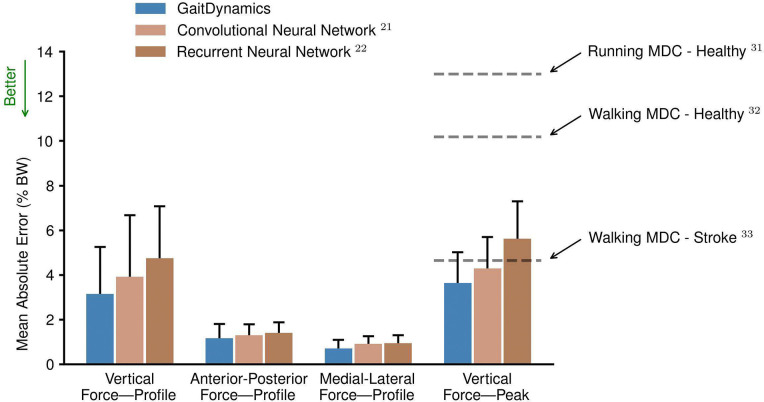
Mean absolute errors of force estimation for the GaitDynamics model and recently published data-driven models when using full-body kinematic inputs. Peak vertical force was computed as the maximum value of the vertical force profile during each gait cycle. The error bars are the mean and one standard deviation across 14 test set studies. The horizontal dashed lines are the minimal detectable changes (MDCs) reported in literature. BW denotes body weight.

The accuracy of GaitDynamics for all force variables surpassed state-of-the-art models that used a convolutional neural network^21^ or a recurrent neural network^22^ (Fig. 1). When using full-body kinematic inputs, the mean absolute errors of GaitDynamics in estimating the vertical force profile, anterior-posterior force profile, medial-lateral force profile, and peak vertical force were 3.2%, 1.2%, and 0.7%, 3.6% Body Weight (BW), respectively. Further, the errors of GaitDynamics in estimating the peak vertical force were smaller than clinically meaningful thresholds—minimal detectable change (MDC)—for both healthy participants (MDCs of 10.2%–13.0% BW)^31,32^ and individuals post-stroke (MDC of 4.7% BW)^33^. This demonstrates that GaitDynamics can identify changes in vertical forces that are greater than measurement errors^34^.

For studies with healthy participants and not used to train the model, mean absolute error ranges were 1.1%–3.9%, 0.8%–1.5%, and 0.5%–0.7% BW for vertical, anterior-posterior, and medial-lateral force profiles, respectively, comparable to those of held-out participants from the training set studies (1.3%–3.3%, 0.4%–1.5%, and 0.3%–2.0% BW; Supplementary Table 1). Although we only used healthy participants’ data to train GaitDynamics, the model maintained its accuracy for individuals with osteoarthritis, whose average mean absolute error were 3.8%, 1.6%, and 0.9% for vertical, anterior-posterior, and medial-lateral force profiles, respectively. However, the accuracy was lower for an individual post-stroke, whose average mean absolute error were 10.1%, 3.1%, and 1.0% BW for vertical, anterior-posterior, and medial-lateral force profiles.

**Fig. 2.**
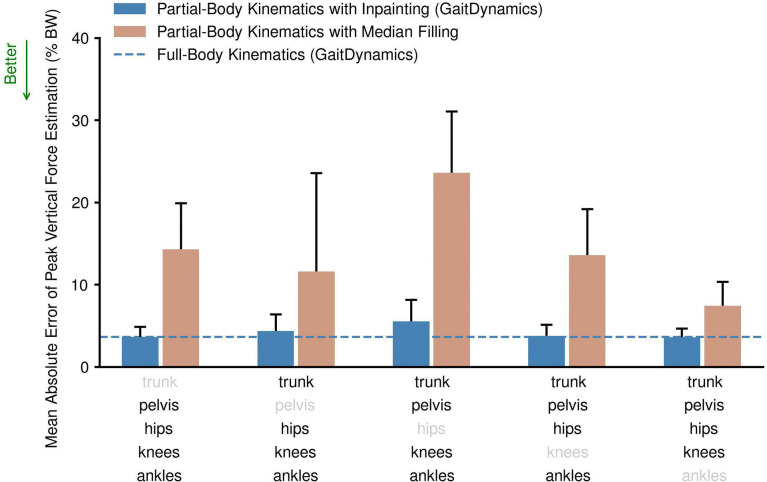
Mean absolute error of peak vertical force estimation when using various combinations of partial-body kinematics as inputs. The light gray text denotes the masked (excluded) kinematic inputs. The bars are the mean and one standard deviation across 14 test set studies. The dashed line is the mean absolute error of GaitDynamics when using full-body kinematics as input. BW denotes body weight.

To investigate the influence of incomplete kinematic inputs on the accuracy of estimating forces, we iteratively masked the kinematics of the trunk, pelvis, hip, knee, and ankle joints from the inputs. Compared to using full-body kinematics as inputs, using partial-body kinematics only slightly decreased the accuracy of GaitDynamics by 0.0–1.9% BW, depending on the joint kinematics that were masked (Fig. 2). The largest accuracy decrease of 1.9% BW occurred when kinematics of both the left and right hip were masked. When the trunk, pelvis, both knees, or both ankles (including subtalar joints) were masked, the accuracy decreases were only 0.0%, 0.8%, 0.1%, and 0.0% BW, respectively. The larger accuracy decrease when masking both hips’ kinematics can be attributed to the greater number of degrees of freedom at the hip, which is three, compared to one to two degrees of freedom for other joints. We also compared the performance of GaitDynamics against a baseline method of accounting for missing kinematics, median filling, where we used the median values of each degree of freedom to fill unknown input kinematics and then used the same refinement model to estimate forces. Compared to using full-body kinematics, filling kinematics of the trunk, pelvis, hip, knee, or ankle with their median values decreased the accuracies by 10.7%, 7.8%, 20.0%, 10.0%, and 3.8% BW, substantially larger than 0.0%–1.9% BW obtained by the inpainting of GaitDynamics. Inpainting can also provide an estimate of the forces; however, we found that a refinement model that is specifically trained for force estimation further improved the accuracy (Supplementary Table 2).

We used heuristics to prune noisy, erroneous, and unusable data, which accounted for approximately 25% trials of the dataset. To evaluate the benefits of the pruning, we compared model performance when trained on all the data versus the data after pruning. Compared to training with all the data, training with data after pruning reduced mean absolute errors from 4.2% to 3.6% BW for peak vertical force estimation and from 0.8% to 0.7% BW for medial-lateral force profile estimation. Training with data after pruning did not change mean absolute errors for estimating vertical or anterior-posterior force profiles.

### Downstream Task 2: predicting knee adduction moment during trunk sway gait without experiments

**Fig. 3.**
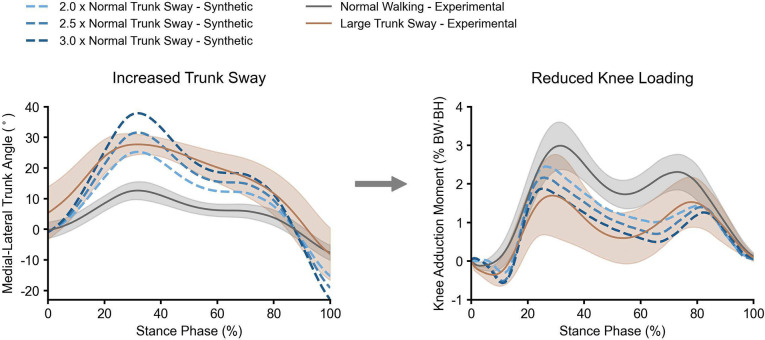
Medial-lateral trunk angles and their corresponding knee adduction moments. Angles and moments are from normal gait^40^ (gray), experimental large trunk sway gait^40^ (brown), and synthetic large trunk sway gait (blue). The shaded areas represent one standard deviation of experimental knee adduction moment. With the increase of synthetic trunk sway angles, the predicted knee adduction moment consistently decreased. BW and BH are body weight and body height, respectively.

Previous controlled experiments identified that increasing medial-lateral trunk sway during walking^35,36^ can reduce knee adduction moments, which are associated with medial compartment knee osteoarthritis^37–39^. We used the GaitDynamics model to replicate the relationship between trunk sway gait and knee loads, without needing experiments. To perform experiment-free prediction, we first used normal walking trials from a test set study^40^ to create synthetic trunk sway gait, increasing medial-lateral trunk angles by 2.0x, 2.5x, and 3.0x of their original magnitudes. We selected 2.0x to 3.0x to encompass values both smaller and larger than the experimental large trunk sway condition (Fig. 3). Then, we input synthetic trunk kinematics along with the remaining kinematics from the normal walking data into the GaitDynamics model to generate full-body kinematics and forces of trunk sway gait. Finally, we computed the knee adduction moment using the cross product of the force vector and the lever arm vector from the knee joint center to the center of pressure.

The knee adduction moments predicted by GaitDynamics were consistently lower as the synthetic trunk sway angles increased from 2x to 3x of normal trunk sway (Fig. 3), which is consistent with prior experiments^40^. The peak trunk sway angles were 13° for experimental normal walking, 28° for experimental large trunk sway gait, and 25°–38° for synthetic large trunk sway gait. The first peak of knee adduction moment reduced by 1.3% Body Weight (BW)⋅Body Height (BH) in experimental measurements, which was larger than 0.5%–1.1% BW⋅BH reductions in synthetic trunk sway gaits generated by GaitDynamics. The second peak of knee adduction moment reduced by 0.8% BW⋅BH in experimental measurements, which was smaller than 0.9%–1.0% BW⋅BH reductions in synthetic trunk sway gaits generated by GaitDynamics. It took less than one second to generate a 1.5-second walking trial on a single Nvidia RTX3050 Laptop graphics processing unit (GPU).

### Downstream Task 3: gait generation at synthetically altered running speeds

Increasing gait speed is a common goal in both rehabilitation and athletics. GaitDynamics can also predict the changes in stride length, peak knee flexion, and peak forces with altered running speeds. We created synthetic anterior-posterior velocities ranging from 3 m/s to 5 m/s by linearly scaling the velocities measured during 4 m/s running^41^ to values between 75% and 125%. Then, we input scaled anterior-posterior velocities along with the remaining kinematics from the 4 m/s experimental data into the GaitDynamics model to generate full-body kinematics and forces of synthetic running speeds.

**Fig. 4.**
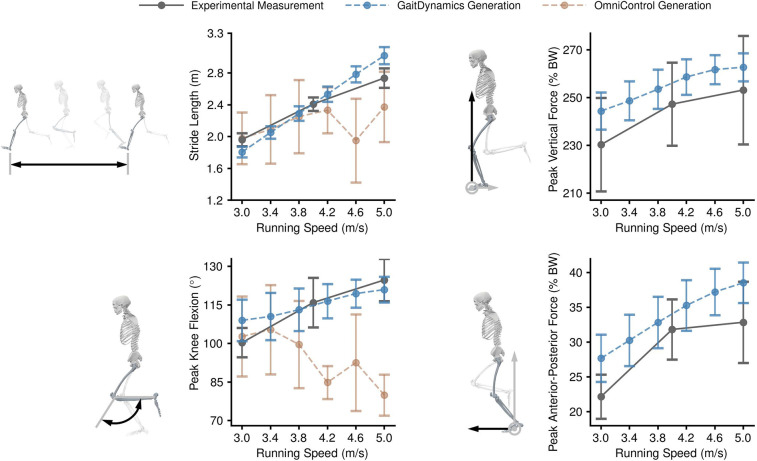
Gait parameters across running speeds. The parameters are measured in experiments^41^ (gray), generated by GaitDynamics (blue), and generated by a state-of-the-art motion generation model, OmniControl^42^ (yellow). The error bars are the mean and one standard deviation. Three out of ten OmniControl generations at a speed of 5 m/s were unsuccessful and thus excluded from the mean and standard deviation calculations. Both kinematic and kinetic parameters generated by GaitDynamics consistently increased with running speed, and their slopes were aligned with those of experimental measurements. In contrast, OmniControl can only generate kinematic parameters.

As running speed increased, GaitDynamics consistently predicted greater stride lengths, peak knee flexion angles, peak vertical forces, and anterior-posterior forces, following the same trend as experimental measurements^41^ (Fig. 4). As running speed increased from 3.0 m/s to 5.0 m/s, there was an expected large increase in the stride length (1.8 m to 3.0 m for GaitDynamics generations vs. 2.0 m to 2.7 m for experimental measurements), as well as a large increase in peak knee flexion angle (109° to 121° for GaitDynamics generations vs. 100° to 125° for experimental measurements). For ground reaction forces, as the running speed increased from 3.0 m/s to 5.0 m/s, there was an expected large increase in the peak anterior-posterior force (39% for GaitDynamics generations vs. 48% for experimental measurements), as well as a much smaller increase in peak vertical force (8% for GaitDynamics generations vs. 10% for experimental measurements). As for the walking generations described above, it took less than one second to generate dynamics of a 1.5-second running trial on a single Nvidia RTX3050 Laptop GPU.

In contrast to GaitDynamics, the state-of-the-art motion generation model, OmniControl, is only capable of generating kinematics parameters^42^. Also, unlike the experimental measurements, the stride length and peak knee flexion generated by OmniControl did not consistently increase at higher synthetic running speeds. In addition, we manually reviewed the OmniControl generations and found that three out of ten generations at a speed of 5 m/s were unsuccessful, as they did not exhibit the characteristics of human running gait (e.g., nearly static posture over time). We removed the three unsuccessful generations from analysis.

## Discussion

We developed the first generative foundation model to quantify gait dynamics and analyze their changes across various gait patterns. GaitDynamics uses a diffusion model, which was able to learn a robust and meaningful representation of gait dynamics encapsulated in the training data. While many existing deep learning models have been proposed for a single gait estimation task, GaitDynamics allows flexible input combinations while maintaining accuracy, positioning it as a foundational model for multiple downstream tasks beyond estimating unknown parameters. We show the utility of GaitDynamics on three diverse downstream tasks, where it successfully predicted (1) forces that drive the movement with full- or partial-body kinematics, (2) the influence of gait modifications on risky knee loading without the need for costly human experiments, and (3) comprehensive kinematic and kinetic changes at a range of gait speeds.

In contrast to previous models developed on small, proprietary datasets, we trained and evaluated GaitDynamics using data from 15 prior studies, aggregating more than 34.8 hours of synchronized kinematics and force data at various gait speeds and patterns. The extensive size and diversity of this dataset enabled GaitDynamics to generalize to unseen studies and populations (i.e., individuals with osteoarthritis) who were not included in the training data. Although the dataset used for developing GaitDynamics is harmonized from high-quality biomechanical studies, noisy and erroneous data still exist in some trials. We used heuristics to prune such data, which helped improve the accuracy of force estimation. Thus, when using open-source datasets to train foundation models for human movement analysis, improving data quality can even outweigh increasing the quantity of data. This observation aligns with practices in contemporary training methods for large language models^43,44^.

For ground reaction force estimation, GaitDynamics achieved smaller errors than state-of-the-art models and was generalizable to studies and participants not used for training (Supplementary Table 1). The training set studies exclusively included healthy participants, so GaitDynamics was not exposed to pathological gait during training. Still, the model maintained its accuracy for individuals with osteoarthritis, though it showed a drop in accuracy for an individual post-stroke. One explanation for the accuracy drop difference is that the individual post-stroke has a substantially lower cadence compared to healthy participants, whereas individuals with osteoarthritis maintained cadences similar to those of healthy participants.

GaitDynamics demonstrated the feasibility of generating forces and joint moments corresponding to synthetic kinematic data altered from normal gait, enabling experiment-free, efficient prediction of gait modification outcomes. GaitDynamics’s ability to predict knee joint moments during trunk sway gait is likely learned from one of the training set studies involving participants walking with varied trunk sway angles^45^. Although the training set participants performed bilateral trunk sway on a treadmill, GaitDynamics successfully predicted knee adduction moment reductions that aligned with those of test set participants performing ipsilateral trunk sway overground.

GaitDynamics can predict interactions between pairs of gait parameters (e.g., joint angle vs. gait speed), potentially making it a valuable tool for more effective coaching and rehabilitation. For example, it can identify parameters that are outside of the distribution of healthy gait, thereby training the user to walk and run more naturally. We demonstrated that kinematic and kinetic parameters generated by GaitDynamics aligned with those of experimental measurements across running speeds. In contrast, a state-of-the-art motion generation model from the computer graphics community, OmniControl, cannot generate kinetic parameters or predict the changes of kinematic parameters at increased running speeds.

GaitDynamics can generate a 1.5-second data window within one second on a personal computer, making it substantially more efficient than physics-based predictive simulations. Recent simulation studies have attempted to accelerate the simulation speed using techniques such as direct collocation and algorithmic differentiation, reducing the simulation time from hundreds of hours^18,19^ to approximately one hour^28,29,46^. Still, GaitDynamics is a thousand-fold more efficient than the accelerated simulation, and thus may enable systematic analyses that were previously infeasible. For example, it may enable systematic investigation of various gait modification strategies or their combinations at a fine-grained resolution, leading to the discovery of new gait modification strategies that maximize clinical outcomes.

Our study has a few limitations. First, as suggested by the name, GaitDynamics may not be applied to non-gait prediction and analysis tasks because its training data was predominantly walking and running gait. Future work may consider incorporating more non-gait data through human experiments, by self-supervised learning methods that do not require force measurements^47–49^, or by simulations that do not rely on human experiments^50,51^. Second, since GaitDynamics does not include muscles, it may not provide muscle-level insights that are available in many simulation studies. Future foundation models could incorporate muscle activation, offering an alternative for computationally intensive musculoskeletal simulations.

In conclusion, GaitDynamics is a generative foundation model that enables robust estimation of forces with flexible combinations of kinematics and can generalize to studies and populations not used to train the model. GaitDynamics’s robustness to incomplete kinematic inputs demonstrated its applicability for in-the-wild scenarios where input sensor data may be incomplete. GaitDynamics can rapidly generate full-body kinematics and kinetics based on synthetic gait parameters, facilitating gait optimization for reduced joint loading or increased running speeds without human experiments. Its accuracy, flexibility, and generalizability can be attributed to the modeling of a large, diverse gait dataset with diverse participant demographics and gait patterns. This study marks a notable advancement in human gait analysis, transitioning away from resource-intensive laboratory-based experiments and simulations. This work will advance the understanding of fundamental principles that govern human movement, potentially facilitating injury prevention, movement rehabilitation, exoskeleton developments, and sports coaching.

## Methods

### Datasets

We trained and evaluated our model using the AddBiomechanics dataset that harmonized marker-based motion capture and force plate data from 15 prior studies^27^. The data were processed by the AddBiomechanics engine^52^, which used marker data to perform scaling of an OpenSim skeletal model without arms^53^ and inverse kinematics to determine the pelvis (root of the skeletal model) positions and joint angles over time. Note that we did not use the force-based kinematics tuning described in the AddBiomechanics paper to prevent the leakage of force data (output of GaitDynamics) into the kinematics (input of GaitDynamics).

We performed the following pre-processing steps to further process the data before using them for model training.

- Of the studies in the AddBiomechanics dataset, eight out of fifteen studies provided kinematics at 100 Hz whereas the remaining seven studies provided kinematics between 120–250 Hz. To minimize distribution discrepancies, we linearly resampled the data from these seven studies to 100 Hz.
- We computed joint angular velocities for all trials in the dataset using finite differences.
- The AddBiomechanics dataset provided 3-D joint angles as Euler angles. However, Euler angles are discontinuous representations of orientation that are more difficult for deep neural networks to learn compared to continuous representations^54^. Thus, we first converted each Euler angle to a 3×3 rotation matrix, and then extracted the first two rows of the matrix to form a 6D vector.
- For each trial, we applied a rotation matrix, which converts the median yaw angle of the pelvis (root of the skeletal model) orientation to zero, to the root orientation of all frames. This made all gait trials independent of the direction in which the skeletal model is facing.
- The dataset included both overground and treadmill gaits, where gait velocities should be measured relative to the Earth and the treadmill belt, respectively. To mitigate this discrepancy, for overground trials, we computed the velocities by finite differencing the positions. For treadmill trials, we first estimated the belt speed by finite differencing anterior-posterior foot position during mid-stance where the vertical force is larger than 40% of body weight. Then, the missing belt speeds between mid-stances were estimated via linear interpolation. Finally, the velocities were estimated as follows:
$${\overset{ˆ}{v}}_{x}=v_{\text{pelvis},x}-\left({\overset{ˆ}{v}}_{\text{right}\;\text{belt}}+{\overset{ˆ}{v}}_{\text{left}\;\text{belt}}\right)/2\;{\overset{ˆ}{v}}_{y}=v_{\text{pelvis},y}\;{\overset{ˆ}{v}}_{z}=v_{\text{pelvis},z}$$
  where $v_{\text{pelvis}}$ denotes the ground-truth pelvis velocities in the Earth frame while ${\overset{ˆ}{v}}_{\text{right}\;\text{belt}}$ and ${\overset{ˆ}{v}}_{\text{left}\;\text{belt}}$ denote the estimated belt speeds.
- We expressed the center of pressure in the calcaneus frame of the stance foot rather than in the Earth frame. Also, when the vertical force is small, noise in the force measurements can cause center of pressure drifts. Therefore, we divided the center of pressure by the height and multiplied it by the magnitude of the vertical force.
  We also performed the following quality check steps to prune noisy, erroneous, or unusable data.
- Due to the non-convex nature of inverse kinematics, a few trials had implausible trunk rotation angles (yaw angle) that were consistently larger than 45°. We pruned 121 trials (0.5%) whose average trunk rotation angles were larger than 45°.
- We aligned the direction in which the skeletal model is facing, as discussed above. However, alignment was infeasible for 1,682 trials (7.5%), because their peak-to-peak changes in moving direction exceeded 45°. Therefore, we pruned these trials.
- Trials shorter than the minimal length of the model input, 1.5 s, cannot be used for model training. We pruned 3,669 trials (16.4%) that were shorter than 1.5 s.
- We noticed that the distances between the center of pressure and the contact foot were unfeasibly large in a few composing studies of the dataset. We pruned 3,725 trials (16.7%) where this distance exceeded 0.3 meters for more than 0.2% of the stance phase.
- For each frame, if the angular velocity of any joint was higher than 2,000 °/s, this frame, its previous 2 frames, and its following 2 frames were pruned from the dataset. The threshold, 2,000 °/s, is a conservative value determined based on maximum hip and knee angular velocities (≤ 800 °/s) in a high-speed (6.5 m/s) running study^55^. There were 833 frames that exceeded this threshold, accounting for fewer than 0.01% of the total frames.

This data pruning process yielded 34.8 hours of data from 270 participants. For model training, we included both gait and non-gait motions such as squat, hopping, and idling. We utilized eight out of fifteen studies in the AddBiomechanics dataset. For each of these eight studies, we used all participants (n=178) except one for training (Table 1). Then, we evaluated the model’s performance on participants (n=92) from two groups: the eight held-out participants from the studies used for training, as well as all participants from the seven studies not used for training. This train-test split allows us to evaluate the model’s generalizability in force estimation across both studies included and not included in the training process. To evaluate the benefits of the proposed data pruning criteria, we compared model performance when trained on data after pruning versus on all the data (except trials shorter than 1.5 s that are not usable).

### GaitDynamics Architecture and Training

**Fig. 5.**
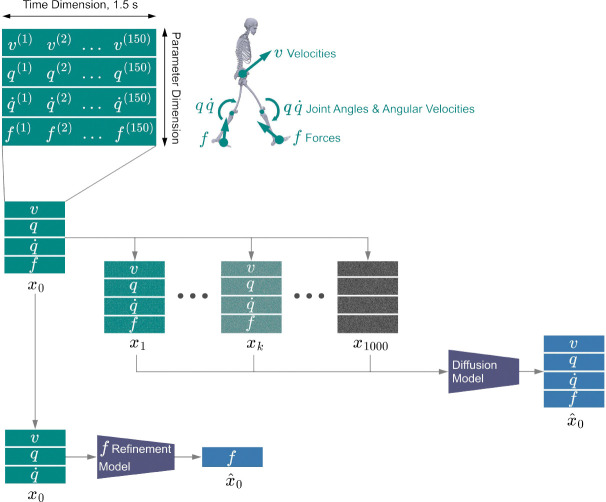
The data window and training of GaitDynamics. The data is a 2-D window with a 1.5-second time dimension and a parameter dimension. The parameter dimension includes velocities v, joint angles q, joint angular velocities $\dot{q}$, and forces f. GaitDynamics has one diffusion model and one force refinement model. For the diffusion model, we followed the definition of diffusion as a Markov chain that gradually adds Gaussian noise to the clean data windows based on step k. At $k=0,x_{0}$ is a clean data window with no noise added, whereas at $k=1000,x_{1000}$ is pure Gaussian noise. A diffusion transformer was trained to recursively restore the clean windows from the windows with different amounts of noise. In addition, a refinement model was separately trained to map full-body kinematics to forces.

GaitDynamics models 2-D data windows, x, which have one parameter dimension and one temporal dimension (Fig. 5). The parameter dimension has 75 elements, corresponding to velocities (v), joint angles (q), and joint angular velocities $\left(\dot{q}\right)$ of an OpenSim model^53^, as well as forces of both feet (f). Each parameter was normalized by min-max normalization, whose bias and scale were determined by the minimum and maximum values of the entire training set data. The temporal dimension consists of 150 time steps, corresponding to 1.5 s of data. This window length is conservatively chosen to exceed typical gait cycle durations.

GaitDynamics consists of one diffusion model and one force refinement model. The diffusion model learns to estimate the data distribution of the human gait using iterative processes of adding noise to the window and denoising the windows. It has a transformer architecture similar to those of prior human kinematics modeling studies^56,57^. The transformer has four self-attention blocks, each configured with 4 attention heads, a 256-dimensional latent space, and 1024 feedforward units. During training, clean data windows were diffused into noisy data windows (Fig. 5) through a Markov noising process as follows:

$$Q\left(x_{k}|x_{0}\right)\sim N\left(\sqrt{a_{k}}x_{0},\left(1-a_{k}\right)I\right),$$

where k∈[0,1000] is the diffusion step, $Q\left(x_{k}|x_{0}\right)$ denotes the probability distribution of noisy window ($x_{k}$) conditioned on the clean data $\left(x_{0}\right),N$ denotes a normal distribution with a mean value of $\sqrt{a_{k}}x_{0}$ and a covariance matrix of $\left(1-a_{k}\right)I,\sqrt{a_{k}}$ are constants following a monotonically decreasing (cosine) schedule for increasing k, and I denotes an identity matrix. At $k=0,x_{0}$ is a clean data window with no noise added, whereas at k=1000, the window $x_{1000}$ is pure Gaussian noise. After adding noise to clean data windows, the diffusion model $\left(\epsilon_{\theta }\right)$ was trained to remove noise from noisy data windows based on k.

We improved the accuracy of force estimation using an end-to-end force refinement model, which takes full-body kinematics as input to estimate ground reaction forces of both feet. The force refinement model has a transformer architecture similar to that of a prior kinematics-based force estimation study^49^. The transformer has six self-attention blocks, each configured with 8 attention heads and 512 feedforward units. For both diffusion model and force refinement model, we used rotary position embedding^58^ along the temporal dimension, 10% dropout, 10^−5^ layer normalization, and rectified linear unit (ReLU) activation function. We used the Adan optimizer^59^ to minimize the mean squared error with a learning rate of 10^−3^, default $\beta (\beta_{1}=0.02,\beta_{2}=0.08,\beta_{3}=0.01)$, and a minibatch size of 256. When sampling windows for each minibatch, we implemented uniform probability sampling across all training set studies and trials to avoid biases from training set studies with larger sizes or from longer trials. We trained the diffusion model and the force refinement model using 1 × 10^5^ and 3 × 10^5^ backpropagation steps, respectively, without early stopping. GaitDynamics was implemented in Python 3.9 with PyTorch 1.13 and Numpy 1.23. The training takes 30 hours on a single NVIDIA RTX A6000 GPU.

### Evaluation

We evaluated GaitDynamics on three downstream tasks: (1) force estimation using various input combinations, (2) knee adduction moment prediction using synthetically increased trunk sway angles, and (3) kinematic and kinetic prediction using synthetic running velocities. We evaluated Downstream Task 1 on the entire test set, including all the participants from studies not used for training as well as all held-out participants from the studies used for training. For Downstream Tasks 2 and 3, we used two studies not used for training. These two studies were the only ones containing experimental measurements for comparison with GaitDynamics generations.

### Downstream Tasks 1: force estimation using flexible combinations of kinematic inputs.

**Fig. 6.**
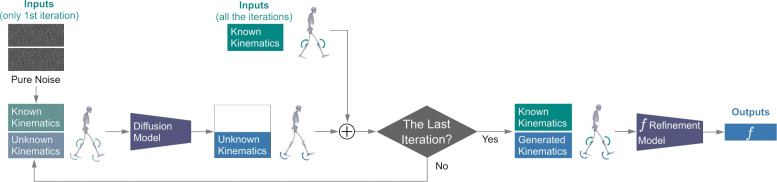
Force estimation using GaitDynamics with partial-body kinematics as input. GaitDynamics generates unknown kinematics using inpainting, where the diffusion model recursively denoise the data window based on the known portion. The inpainting begins by denoising pure Gaussian noise. Then, the denoised data window (unknown kinematics only) is concatenated with known kinematics and diffused to the noisy data window of the next iteration following a Markov chain. Subsequently, the same generation, concatenation, and diffusion processes are iteratively applied to the data window for a total of 50 iterations, where the quality of generation gradually increases. Finally, the unknown kinematics generated in the last iteration are concatenated with known kinematics and fed into the refinement model to estimate forces.

For partial-body kinematics, GaitDynamics first uses the diffusion model to generate unknown kinematics, followed by a refinement model that maps full-body kinematics to forces (Fig. 6). The diffusion model generates unknown kinematics using the Denoising Diffusion Implicit Models (DDIM) sampling^60^ through a process called inpainting^61^, where the unknown portion of the data window is recursively denoised based on the known portion. Specifically, the diffusion starts by initializing the noisy window $x_{k}$ as pure Gaussian noise and denoising it as follows:

$$\begin{array}{c} {\overset{ˆ}{x}}_{0}=\frac{x_{k}\;-\;\sqrt{1\;-\;\alpha_{k}}\epsilon_{\theta }\left(x_{k}\right)}{\sqrt{\alpha_{k}}}\\ k=1000, \end{array},$$

where ${\overset{ˆ}{x}}_{0}$ denotes the denoised window of the current iteration, $\alpha_{k}$ denotes a constant following the noise scheduler, $\epsilon_{\theta }$ denotes the diffusion model, and 1000 is the diffusion step of the first iteration. Next, the unknown portion of the denoised data window is concatenated with the known kinematics. Then, the concatenated window is diffused following a Markov chain to create the noisy data window of the next iteration. The denoising, concatenation, and diffusion processes are recursively applied to the data window until the last iteration, at which point the diffusion iteration is complete and the diffusion model generation is obtained. We performed DDIM sampling with 50 iterations, with each iteration advancing 20 diffusion steps (1000/50). Finally, the generated kinematics along with the known kinematics are provided as inputs into the refinement model for force estimation.

To evaluate various combinations of partial-body kinematics, we iteratively masked kinematics of trunk, pelvis, hip, knee, and ankle joints from inputs, used inpainting to generate masked kinematics, and estimated forces using the refinement model. We also implemented a baseline method for filling masked kinematics, Median Filling, where the unknown kinematics are filled by its median value. Median Filling used the same force refinement model as inpainting for force estimation.

We compared the performance of GaitDynamics in force estimation against two state-of-the-art models specifically designed to use joint kinematics for force estimation. Their architectures—convolutional neural network^21^ and recurrent neural network^22^—are representative architectures distinct from the transformers used in GaitDynamics. For both models, we implemented their model architectures and used the same dataset, optimizer, batch size, and learning rate as those of GaitDynamics. Based on the lowest training set loss, we trained the convolutional and recurrent models with 3 × 10^5^ and 0.79 × 10^5^ backpropagation steps, respectively.

We exclusively evaluated GaitDynamics’ force estimation performance on gait data of the test set. First, we excluded trials if their names explicitly contain non-gait terms such as “jumping”, “squat”, “sit”, etc. Fourteen out of the fifteen studies in the AddBiomechanics dataset were retained, with one study excluded due to the absence of gait trials. Second, we segmented the continuous gait data into individual gait cycles using a two-step process: (1) identifying tentative left foot gait cycles (from heel-strike to heel-strike) using vertical force with a threshold of 10% body weight and (2) excluding gait cycles whose lengths are not within 0.2–2 s or their stance phase lengths are not within 0.1–1.5 s. We used these conservative thresholds for gait cycle lengths to minimize the probability of excluding valid walking or running gait data. Still, we found that one of the test set studies has an individual post-stroke whose gait cycles were substantially longer than these thresholds. Thus, for this particular study, we adjusted the length thresholds for gait cycles and stance phases to 0.1–3 s and 0.2–4 s, respectively. The peak vertical force was computed as the maximum vertical force of a gait cycle. We calculated the mean absolute error by averaging all the gait cycles within each study. We then computed the mean and one standard deviation of fourteen studies’ errors, ensuring that studies of different sizes contributed equally to the metrics.

### Downstream Tasks 2: predicting knee adduction moment during trunk sway gait without experiments.

**Fig. 7.**
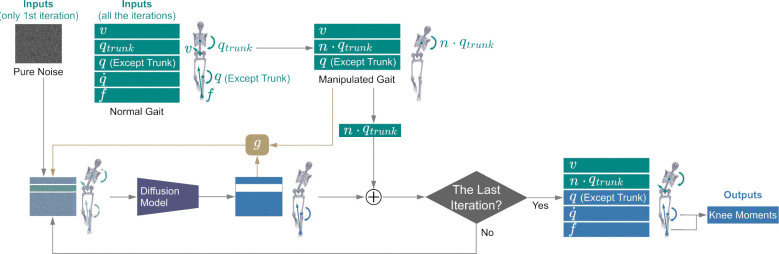
Experiment-free prediction of knee adduction moment during walking with large medial-lateral trunk sway angles. The process is similar to the inpainting described in Fig. 6, with two differences. First, instead of using experimental data as input, manipulated gait data with n times larger medial-lateral trunk sway are used as input. Second, a loss function, g, is used to regularize large discrepancies in v and q for each iteration. Discrepancies larger than a threshold are back-propagated to refine the noisy motion using gradient descent optimization. Finally, based on the generated q (except trunk) and $\overset{‾}{f}$, we computed the knee adduction moment using the cross product of the force vector and the lever arm vector from the knee joint center to the center of pressure.

Only one training set study includes trunk sway gait, where participants walked with increased bilateral trunk sway on a treadmill^45^. We performed testing on one test-set study^40^ that was not used for model training. The study involves ten participants performing normal walking and walking with increased ipsilateral trunk sway, leaning towards the left foot. Thus, we performed the analyses on each participant’s left knee. For all the normal walking trials, we created manipulated gait by amplifying the medial-lateral trunk angles to 2.0x, 2.5x, and 3.0x of their original values while retaining the original values of other kinematic parameters (Fig. 7). Then, based on the manipulated trunk kinematics $n\cdot q_{trunk}$, we generated unknown kinematics and forces using DDIM sampling and inpainting described in Downstream Task 1. To ensure the model generated walking gaits, we used a loss function g to regularize large discrepancies between the manipulated gait window and the denoised window $\left({\overset{ˆ}{x}}_{0}\right)$ in each iteration as follows:

$$\begin{array}{c} g=SUM(ReLU(|{DoF}_{i}-{\hat{DoF}}_{i}|-10\%\cdot {RoM}_{i})),\\ {DoF}_{i}\in v,q \end{array}$$

where ReLU is a Rectified Linear Unit function, ${DoF}_{i}$ denotes each degree of freedom of v and q, and ${RoM}_{i}$ is the range of motion for the corresponding degree of freedom. As such, we did not regularize any discrepancies in joint angular velocities or forces, nor small discrepancies in velocities or joint angles that were within 10% of the range of motion. During each inpainting iteration, the loss function g was back-propagated to the noisy data window five times, iteratively adjusting the window using gradient descent optimization with a learning rate of 0.02. For both experimental measurements and GaitDynamics generations, we calculated knee adduction moments via the cross-product approach^62^ as follows:

$$\text{Knee}\;\text{Adduction}\;\text{Moment}=\frac{r_{y}\;\cdot \;f_{z}-r_{z}\;\cdot \;f_{y}}{\text{Body}\;\text{Weight}\;\cdot \;\text{Body}\;\text{Height}},$$

where r denotes the lever arm vector from the knee joint center to the center of pressure, and y and z are aligned with vertical and medial-lateral direction, respectively.

### Downstream Tasks 3: gait generation at various running speeds.

Three training set studies include running trials with speeds ranging from 2.3 m/s to 4.7 m/s^63–65^. We performed testing on one test-set running study^41^ that was not used for model training. The study involves ten participants performing normal running on a treadmill at four different speeds, i.e., 2.0 m/s, 3.0 m/s, 4.0 m/s, and 5.0 m/s. Following the original study, we performed the analyses on each participant’s right-foot gait cycles, which were segmented using vertical force with a threshold of 10% BW. For each participant’s experimental 4.0 m/s running data, we scaled anterior-posterior velocities by factors from 75% to 125% in 10% increments, thus creating manipulated running speeds of 3.0 m/s to 5.0 m/s in 0.4 m/s increments. We retained vertical and medial-lateral velocities of experimental 4.0 m/s running. We applied the method used in Downstream Task 2 (Fig. 7) to generate full-body kinematics that were coherent to the manipulated running speeds. The known parameters of this task were manipulated velocities, instead of trunk kinematics that were used in Task 2. To generate a wide range of running speeds, the loss function g only regularizes discrepancies in velocities v or joint angles q when they exceed 30% of the range of motion.

We compared the performance of GaitDynamics in kinematics generation against a state-of-the-art motion generation model, OmniControl, which uses partial-body kinematics and text as inputs^42^. We implemented OmniControl by directly using its open-source code and trained model. The kinematic inputs were pelvis (root of the skeletal model) positions computed from running speeds of 3.0 m/s to 5.0 m/s in 0.4 m/s increments. The text input was “a person runs continuously in a straight line”, consistent for all the running speeds. We did not specify the numerical value of speed in the text inputs since they led to more unsuccessful generations in pilot testing. We repeated the generation ten times with different random seeds. We segmented the gait cycles of the right foot using its anterior-posterior velocity with a threshold of 0.1 m/s. We manually reviewed generations with no gait cycle detected and removed the generation if we confirmed that the generation was unsuccessful (i.e., not human running gait). For the successful generations, we first computed the average stride length and peak knee flexion angle across gait cycles within each generation, and then computed the mean and one standard deviation across generations of each speed.

## Supplementary Material

Supplement 1

## Figures and Tables

**Table 1.** Heights and weights of training and test set participants.

	# of studies	# of participants	# of trials	Height	Weight

Training	8	178	10,352	1.71 ± 0.09 m	68.1 ± 11.8 Kg
Test	15*	92	1,929	1.67 ± 0.17 m	63.6 ± 17.3 Kg

* The 15 studies include 8 studies used for training; however, testing was performed on held-out participants (not used for training) from these studies.

## Data Availability

This work utilized the AddBiomechanics dataset that consists of data from fifteen prior studies (https://addbiomechanics.org/download_data.html).
