# Supplementary material for: GaitDynamics: A Generative Foundation Model for Analyzing Human Walking and Running
Source: Res Sq. 2025 Mar 21:rs.3.rs-6206222. Preprint. [Version 1] doi: 10.21203/rs.3.rs-6206222/v1 (PMC11957236; doi:10.21203/rs.3.rs-6206222/v1)
Supplement: Supplement 1 [file NIHPPrs6206222v1-supplement-1.pdf]

Supplementary

Supplementary Table 1. Mean absolute error of the GaitDynamics on vertical (f_v), anterior-posterior (f_{ap}), and medial-lateral (f_{ml}) force profile estimation for 14 composing studies of the AddBiomechanics dataset.

Study	Used for Training	Health Status	Activity	Terrain	Mean Absolute Error (%Body Weight)		
					f_v	f_{ap}	f_{ml}
Camargo2021	✓	Healthy	Walking	Overground + Treadmill	3.3	1	0.7
Moore2015	✓	Healthy	Walking	Treadmill	2.2	0.9	0.6
Tan2022	✓	Healthy	Walking	Treadmill	1.3	0.4	0.3
vanderZee2022	✓	Healthy	Walking	Treadmill	3.1	0.7	0.5
Wang2023	✓	Healthy	Walking & Running	Treadmill	2.9	0.8	0.7
Carter2023	✓	Healthy	Running	Treadmill	1.7	1.5	2.0
Tan2021	✓	Healthy	Running	Treadmill	1.8	0.6	0.5
Falisse2017	×	Healthy	Walking	Overground	3.1	1.5	0.5
Han2023	×	Healthy	Walking	Overground	2.6	0.9	0.5
Lencioni2019	×	Healthy	Walking	Overground	1.1	0.8	0.5
Uhlrich2023	×	Healthy	Walking	Overground	3.9	1.3	0.6
Hamner2013	×	Healthy	Running	Treadmill	3.1	1.4	0.7
Fregly2012	×	Osteoarthritis	Walking	Overground	3.8	1.6	0.9
Li2021	×	Stroke	Walking	Treadmill	10.1	3.1	1.0
Average and one standard deviation					3.2 ± 2.1	1.2 ± 0.6	0.7 ± 0.4

Note that for each study used for training, the mean absolute errors were from the participant held out from training.

Supplementary Table 2. Mean absolute error of vertical (f_v), anterior-posterior (f_{ap}), and medial-lateral (f_{ml}) force profile estimation when using only the diffusion model with inpainting versus using GaitDynamics with both diffusion and refinement models.

Input Kinematics					Mean Absolute Error (%Body Weight)					
					Diffusion Only			GaitDyanmics (Diffusion & Refinement)		
Trunk	Pelvis	Hips	Knees	Ankles & Subtalar	f_v	f_{ap}	f_{ml}	f_v	f_{ap}	f_{ml}
×	✓	✓	✓	✓	4.9 ± 3.0	1.5 ± 0.6	1.0 ± 0.5	3.1 ± 1.6	1.4 ± 0.5	0.7 ± 0.3
✓	×	✓	✓	✓	5.7 ± 5.5	1.5 ± 0.5	1.0 ± 0.4	4.0 ± 3.9	1.4 ± 0.5	0.9 ± 0.4
✓	✓	×	✓	✓	6.5 ± 5.8	1.6 ± 0.5	1.3 ± 0.5	5.1 ± 4.3	1.6 ± 0.6	1.4 ± 0.4
✓	✓	✓	×	✓	5.1 ± 3.9	1.5 ± 0.5	0.9 ± 0.3	3.4 ± 2.5	1.3 ± 0.6	0.7 ± 0.4
✓	✓	✓	✓	×	6.0 ± 4.9	1.6 ± 0.6	1.0 ± 0.3	3.1 ± 1.4	1.3 ± 0.5	0.8 ± 0.4

Errors are reported as mean and one standard deviation of 14 composing studies of the AddBiomechanics dataset.
